# VRP in urban areas to optimize costs while mitigating environmental impact

**DOI:** 10.1007/s00500-022-07325-z

**Published:** 2022-07-22

**Authors:** Carmine Cerrone, Anna Sciomachen

**Affiliations:** Department of Economics and Business Studies, University of Genoa, Via Vivaldi 5, 16126 Genoa, Italy

**Keywords:** Green vehicle routing, Urban distribution, Meta-heuristic

## Abstract

Nowadays, the need to think about sustainable mobility, both goods and people, is widely recognized. For this reason, many recent papers have moved in this direction. In this context, particular attention is now devoted to urban mobility, mainly from a smart city perspective. The present work focuses on sustainable urban freight distribution and proposes a variant of the VRP, which presents some innovative aspects. The goal is to minimize the routes’ cost components, including traveling and external costs due to environmental issues, depending on the chosen vehicles and the different urban streets to cross. In addition, restrictions on the maximum duration of each route to ensure frequent sanitation of vehicles used for deliveries, as required from the beginning of the COVID-19 pandemic, are imposed. The distribution network is modeled by a weighted digraph for which some properties are proved. To face the problem, we present a mixed-integer linear programming model, a math-heuristic associated with it, and a memetic algorithm approach. The results of the reported computational experimentation with random instances specifically tailored for the problem show the efficiency of the proposed methods. Further, test cases based on data of the distribution network of two B2C companies operating in the city of Genoa, Italy, proved the effective application of the proposed methods in the direction of sustainable urban distribution plans.

## Introduction

From the last few years, almost daily, media reports of damage caused by weather and atmospheric phenomena. Public opinion agrees on the need to change their lifestyles towards more environmentally sustainable behavior, while both scientists and politicians call for a reduction of pollutants to protect the environment. Above all, logistics and transport enterprises cannot ignore the need to establish shipping and routing plans aimed at reducing, among others, emissions, noise, and accidents along their paths. In this direction, recent literature has presented many works, having as one of the main goals the minimization of the mentioned above external costs and their evaluation. More precisely, some papers in the last few years proposed either different ways to calculate and evaluate externalities (see, e.g., Bektaş et al. 2019; Bigazzi and Figliozzi 2013; Dekker et al. 2012; Petro and Konečnỳ 2019) or pricing policies to reduce them (see, among others, Kellner et al. 2017; Chang et al. 2018). Other research works presented strategies to incentivize vehicles or transport modalities with lower environmental impact (Okushima 2018; Xu et al. 2019). In particular, taking into account also the social need and the impact that distribution of goods has on citizens, in the current decade, many studies have been proposed aimed at sustainable urban mobility, especially from a smart city perspective (Carrabs et al. 2017; Kauf 2019; Szymczyk and Kadłubek 2019; Strale 2019).

It is true that smart mobility is a crucial topic able to impact the well-being of citizens and the environmental policies of the cities (Taniguchi et al. 2012). As part of the policies implemented in the context of smart mobility to reduce the environmental impact, the distribution of goods, and more precisely the home delivery due to e-commerce, has focused a lot of attention. The goal is twofold: to analyze the likely impact of distribution operations in urban areas upon pollution and to see how sustainable freight distribution can be assumed (Figliozzi 2010; Cerulli et al. 2018; Heshmati et al. 2019).

From an operations research and management science point of view, the Vehicle Routing Problem (VRP) is widely used in decision-making processes about the distribution of goods and services. Today, models and solution methods of VRP are encountered very frequently in the optimization of distribution plans where sustainable factors have to be taken into proper account (Bektaş and Laporte 2011; Behnke and Kirschstein 2017; Demir et al. 2014; Ehmke et al. 2018; Erdoğan and Miller-Hooks 2012; Koyuncu and Yavuz 2019; Kramer et al. 2015; Yu et al. 2019). In the very recent literature, VRPs have been proposed in which, in addition to the need to plan the distribution of goods in urban areas in a sustainable manner, they also consider the need to contain the spread of the COVID-19 virus (Chen et al. 2020; Cerrone et al. 2021). In accordance with this topic, the present work focuses on sustainable urban freight distribution within a smart city framework and proposes a variant of the Green VRP (GVRP), in which mobility models are suggested by the needs arising from the COVID-19 pandemic are also taken into accounts. In particular, in the proposed GVRP, we have to choose from an available fleet of non-homogeneous vehicles, those vehicles to deliver goods of a large-scale retailer company in an urban area. Vehicles differ for their size, capacity, and pollution class. In addition, government traffic restrictions and street limitations are given. The goal is to minimize the cost components, which include traveling, loading, and external costs, due to environmental issues, depending on the type of chosen vehicles and urban streets to cross. The present work originates from and extends the results presented in Carrabs et al. (2017), where the authors proposed a Mixed Integer Linear Programming (MILP) model for defining delivery plans in a Business to Consumer (B2C) context. More precisely, we present a refinement of the model given in Carrabs et al. (2017), by including valid inequalities and examining additional constraints, depending on the vehicle type and EURO-Class. The minimization of emissions and pollution reduction is the main objective or part of the generalized cost function in many VRPs. In literature, these problems have often been called Pollution Routing Problem (PRP): Erdogan and Miller-Hooks (2012), Xiao et al. (2012). In this paper, we propose two different approaches derived from the recent literature to solve our GVRP separating two of the most crucial aspect of any optimization technique: the improving phase and the feasibility restoring.

The organization of the paper is as follows. In Sect. 2 we introduce in detail the underlying optimization problem together with the required notation. Some properties concerning the derived network model are also given. In Sect. 3, we give the formulation of the related MILP model. In Sect. 4 the proposed heuristics are given. Section 5 presents the computational experimentations, based on both random instances and real size instances derived from a retail company located in Genoa, Italy. Finally, we give some conclusions and outline future work.

## Problem definition

As already mentioned, in the present work, we are involved with the definition of sustainable distribution plans of goods in urban areas. This problem can be considered as a variant of the so-called GVRP. More precisely, given a number of customers located in a metropolitan area requiring a known amount of goods and a fleet of not homogeneous vehicles, we have to determine i) a set of routes originating and terminating at a depot and servicing all the customers and ii) which vehicles to select for traveling along the routes. Vehicles differ for their size, capacity, cost, and pollution class. The goal is to minimize the total cost given by traveling, loading, and pollutant cost components. Note that this goal aims at favoring the use of the most eco-friendly vehicles, especially in the city center and close to schools and parks (Carrabs et al. 2014).

To model the problem we are focused on, a digraph $G = (N,A)$ defines the underlying urban transportation network, where $N = \{d\} \cup U$ is the set of $n+1$ nodes, representing the set *U* of customers and the depot node *d*; *A* is the set of *m* paths of the city connecting pair of nodes.

For each customer $i \in U$, we indicate with $q_i$ the required amount of units of goods. A value $l_{ij}$ is associated with each arc $(i, j) \in A$, representing the length of the corresponding road connections.

Set *V* contains the fleet of vehicles. To each vehicle, $v \in V$ has associated a quintuple of parameters (*w*, *e*, *k*, *c*, *s*) specifying, respectively, its class of weight, pollution emission type, capacity, cost/km, and setup cost. All vehicles are grouped into classes *t*, such that given two vehicles $v_i, v_j \in V$, $t_ {v_i} = t_ {v_j}$ if and only if $w_ {v_i} = w_ {v_j}, e_ {v_i} = e_ {v_j}, k_ {v_i} = k_ {v_j}, c_ {v_i} = c_ {v_j}, s_ {v_i} = s_ {v_j}$.

Note that the class of weight of the vehicles determines the subset of urban paths that are inhibited to them, while the type refers to their category in terms of polluting emissions. Finally, note that the cost of each vehicle depends on both its class of weight and the pollution emission type, which refers to the standard EURO-classes from III to VI (see Sect. 2.1).

The solution to the problem is the set of minimum-cost vehicle routes for delivering the required goods to all customers. As in the classical VRP, some conditions have to be satisfied: (i) all nodes, except node *d*, must be served only once by a vehicle; (ii) all vehicles return to node *d* after completing their service; (iii) all vehicles have a maximum capacity $k_v$.

It should be noted that in this work, the time windows for the delivery of goods are given implicitly. In fact, the delivery plans are defined considering only the time slots of a day in which in the city of reference is allowed the transit of commercial vehicles. In particular, the time slots available for the delivery of goods vary from 2 to 4 h depending on the parameters *e* and *w* associated with each vehicle $v \in V$. Thus, let $L_v$ be the maximum length of a tour traveled by vehicle $v, \forall v \in V$, depending on the dimension of the time slot and the parameters $w_v$ and $e_v$. In order to obtain a feasible solution, it is important to take into account the service time of each customer. Considering the average speed of the vehicles, in this paper, the service time is transformed into a distance value $L_c$, so that it can be included in the constraint on the maximum allowed length $L_v$ of each tour. Furthermore, during this period of the COVID-19 pandemic, the presence of these constraints allows the sanitation of the vehicle (and drivers) several times during the day.

### Cost of arcs

In this work, depending on the pollution emission type *e* of each vehicle *v*, the air pollution ($a_v$) is quantified economically, and its impact is added to the cost of each arc in the graph, also considering the marginal climate change costs ($m_v$). We calculate the costs of the arcs, according to the results shown in Cerulli et al. (2018). In particular, the air pollution and marginal climate change costs are considered with respect to three different cases, corresponding to EURO classes III, IV, and V–VI of the vehicles, respectively. The costs due to air pollution and marginal climate change of the fleet of vehicles expressed in Euro/km are reported in Table 1, and refer to the two main types of commercial vehicles, namely light commercial vehicles (LCV) and heavy good vehicle (HGV). The costs reported in Table 1 have been derived from (Ricardo 2014).

In this scenario the cost of crossing arc $(i, j) \in A$ by vehicle *v* is calculated as:

1 $$\begin{aligned} c_{ijv}=(c_v+a_v+m_v)l_{ij}\delta _{ij}. \end{aligned}$$

The parameter $\delta _{ij} \ge 1$ is associated with each arc $(i,j)\in A$ and is used to increase the traversing cost of a street that passes too close to schools, parks, or other places of interest.

**Fig. 1:**
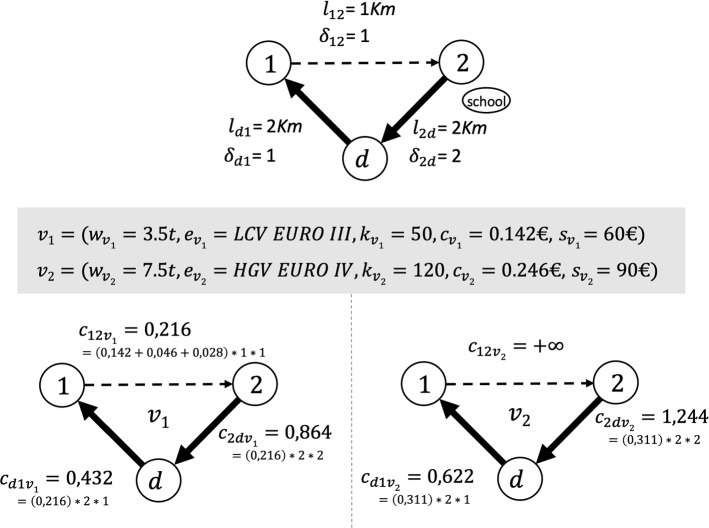
Example of calculation of the arcs’ cost for two vehicles $v_1$ and $v_2$. Dashed lines represent streets that cannot be traveled by HGV vehicles. Therefore, arc (1, 2) is not compatible with the weight class $w_{v_2}$. $\delta _{2d}$ is set to 2 since arc (2, *d*) is adjacent to a school


In many real-world scenarios, crossing a road by vehicles with a specific class of weight (*w*) could be prohibited (see Fig. 1). Considering that a cost $c_ {ijv}$ is associated with each vehicle $v \in V$, in case the road is prohibited for *v*, logically we set $c_ {ijv} = +\infty$ (see Fig. 1). Obviously in all the procedures implemented in this work, arcs with cost $c_ {ijv} = +\infty$ are not created.

**Table 1:** Environmental cost per Euro/Km of the available vehicles

	Air pollution cost $a_v$		Marginal climate change cost $m_v$	
	LCV	HGV-1	LCV	HGV-1
EURO III	0.046	0.061	0.028	0.029
EURO IV	0.032	0.038	0.028	0.027
EURO V–VI	0.011	0.017	0.028	0.025

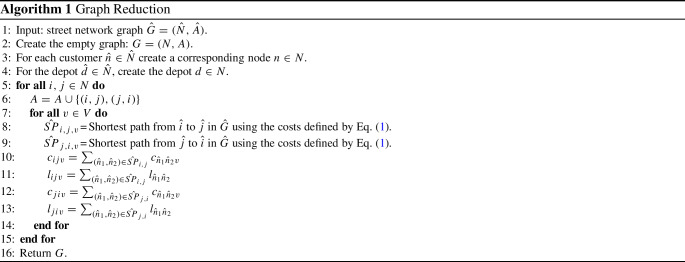


### Graph reduction

Graph $G = (N, A)$ represents the road network within which our vehicles move. In Sect. 2 we have specified that the set *N* of nodes is equal to $\{d\}\cup U$. This implies that excluding the depot *d*, all the nodes of the graph represent customers, and there are no transit nodes.

To transform a generic street network $\hat{G} =(\hat{N},\hat{A})$ in graph *G*, we use the following procedure (see Algorithm 1 - Graph Reduction).

**Fig. 2:**
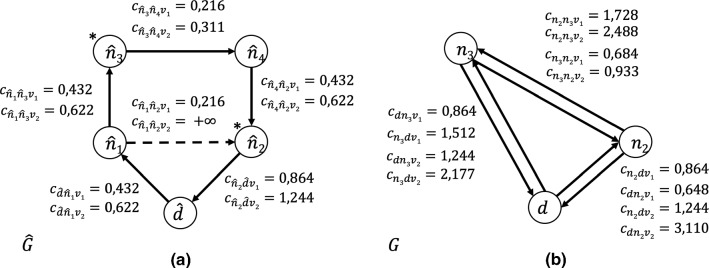
An example of the Graph Reduction Algorithm. In *G* the cost $c_{dn_2v_1}$ of crossing arc $(d,n_2) \in A$ with vehicle $v_1$, represents in $\hat{G}$ the cost of the shortest path $(\hat{d},\hat{n_1})(\hat{n_1},\hat{n_2})$ using the same vehicle $v_1$. The cost $c_{dn_2v_2}$ of crossing arc $(d,n_2) \in A$ with vehicle $v_2$, represents in $\hat{G}$ the cost of the shortest path $(\hat{d},\hat{n_1})(\hat{n_1},\hat{n_3})(\hat{n_3},\hat{n_4}) (\hat{n_4},\hat{n_2})$ using vehicle $v_2$

In lines 4 and 5 of the algorithm, according to the customers and the depot of $\hat{G}$, the corresponding nodes are created in *G*. At line 6, for each pair of nodes $i,j \in N$ the arcs (*i*, *j*) and (*j*, *i*) are created in *A*. On lines 8 and 9 the shortest paths between nodes $\hat{i},\hat{j} \in \hat{N}$ related to nodes $i,j \in N$ are computed. On lines 10 and 12, the cost of each arc $(i,j) \in A$ is created in relation to the specific vehicle *v*. Finally, lines 11 and 13 show the procedure for calculating the route length of the paths between each pair of nodes in *G* on the basis of the average speed of the vehicles and the service time at the nodes, as described above.

**Fig. 3:**
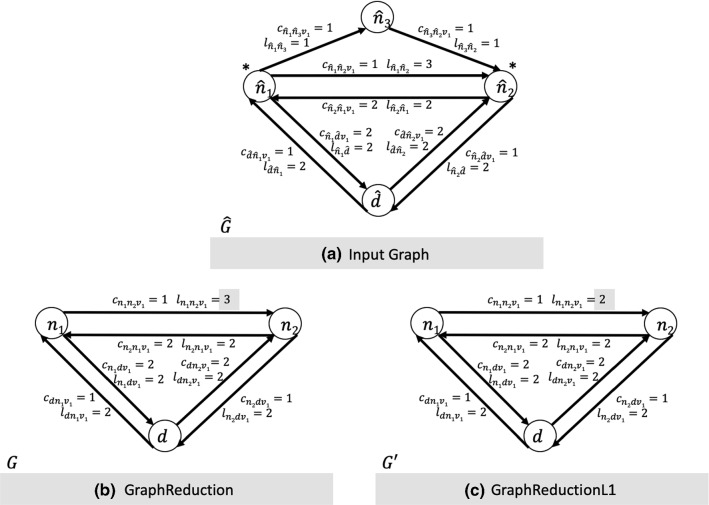
**a** Street network graph; nodes $\hat{n_1}$ and $\hat{n_2}$ are customers. Maximum length imposed for each route equal to 6. $\hat{S} = (\hat{d}, \hat{n_1},\hat{n_3}, \hat{n_2})$ optimal solution in $\hat{G}, \mathrm{cost}=4, \mathrm{length}=6$. **b** Graph Reduction Algorithm example from graph $\hat{G}$ to graph *G*. $S = (d, n_2,n_1)$ optimal solution in $G, \mathrm{cost}=6, \mathrm{length}=6$. **c** Graph Reduction L1 Algorithm example from graph $\hat{G}$ to graph $G'$. $S = (d, n_1,n_2)$ optimal solution in $G, \mathrm{cost}=3, \mathrm{length}=6$

Figure 2 reports an example of how the graph reduction algorithm works. The nodes of the street network $\hat{G}$ (Fig. 2a) are the depot *d*, the customers $\hat{n}_2$ and $\hat{n}_3$, and the transit nodes $\hat{n}_1$ and $\hat{n}_4$. The dashed arc $(\hat{n}_1,\hat{n}_3)$ represents a street prohibited to the transit of HGV vehicles. The weights on the arcs of $\hat{G}$ are those derived in Fig. 1. Figure 2b depicts the resulting graph *G*, which is our referring network model. Arcs of *G* are the solution of the shortest path problem between customers and customers and the depot, according to lines 8 and 9 of the Graph Reduction algorithm. Note that the cost of the path from the depot to customer $n_2$ traveled by vehicle $v_2$ is much higher than the same path traveled by vehicle $v_1$, due to the traffic limitation of graph $\hat{G}$.

### Graph reduction property

The graph reduction function allows us to process smaller inputs speeding up the solution approaches. This section will analyze the impact of the transformation on the optimal solution produced by an exact approach.

#### Proposition 1

Let $\hat{G}$ be the graph of the road network and $G=GraphReduction(\hat{G})$ the reduced graph obtained by applying the algorithm (Graph Reduction). The value of the optimal solution calculated on graph *G* is greater than or equal to the value of the optimal solution calculated on graph $\hat{G}$.

#### Proof

Starting from a solution *S* in *G* it is always possible to create a solution $\hat{S}$ in $\hat{G}$ with the same objective function value. The assumption is trivial since each route $R \in S$ can be transformed into a route $\hat{R} \in \hat{S}$ by replacing the arcs of *R* with the corresponding shortest paths in $\hat{G}$, thus keeping the cost of the route, its travel time and its total demand unchanged. This assures that it is not possible to create a solution in *G* better than the optimal solution presented in $\hat{G}$. To prove this, we will use the example reported in Fig. 3b. $\square$

#### Proposition 2

Let $\hat{G}$ be the graph of the road network and $G=GraphReduction(\hat{G})$ the reduced graph obtained by applying the algorithm (Graph Reduction). If the cost and the length of each arc are such that:

2 $$\begin{aligned} c_{ijv}\le c_{hkv} \Rightarrow l_{ij}\le l_{hk} \ \ \forall (i,j),(h,k) \in \hat{A},\ \forall v \in V \end{aligned}$$

then the cost of the optimal solution calculated on graph *G* is equal to the cost of the optimal solution calculated on graph $\hat{G}$.

#### Proof

Given $\hat{S}$ a feasible solution of our problem on graph $\hat{G}$, let $\hat{R}$ be a sequence of nodes representing a generic route belonging to $\hat{S}$ associated with a generic vehicle $v \in V$. Removing from $\hat{R}$ all nodes with null demand, we will get a new sequence of nodes *R*. Considering that by construction, between each pair of successive nodes in *R* there is a path in $\hat{G}$, so *R* is a route also in *G*. Given two consecutive nodes $(n_i, n_j)$ in *R*, in $\hat{R}$ there is the path composed of nodes $(n_i, \ldots , n_j)$. Further, since each arc of *G* represents the minimum cost path between the same pair of nodes in $\hat{G}$, then the cost $c_ {i j v}$ of the arc $(n_i,n_j)$ traveled with vehicle $v \in V$ is less than or equal to the cost of the route from node $n_i$ to node $n_j$ in $\hat{G}$ with the same vehicle. This means that the cost of the route *R* will always be less than or equal to the cost of the route $\hat{R}$. Based on inequality (2) we can say that the travel time of the arc $(n_i,n_j)$ is less than or equal to the travel time of the path from node $n_i$ to node $n_j$ in $\hat{G}$.

This means that, according to the given travel time constraint, the length (travel time) of route *R* will be less than or equal to that of route $\hat{R}$. Since routes $\hat{R}$ and *R* have the same customer demand, the maximum capacity of the vehicle $v \in V$ is never exceeded. With this simple conversion we have shown that it is possible to transform each solution $\hat{S}$ in $\hat{G}$ into a solution *S* in *G* having a value less or equal to the objective function.

Now using proposition (2.3) our proof is complete. $\square$


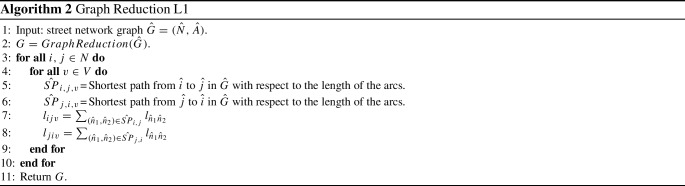


Starting from the *GraphReduction* algorithm, we created the *GraphReductionL*1 algorithm by changing the calculation of the length of the arcs in *G*. In particular $\forall (i,j) \in A, \ \forall v \in V, l_{ilv}$ = minimum path between *i* and *j* in $\hat{G}$ with respect to the length of the arcs in $\hat{A}$.

#### Proposition 3

Let $\hat{G}$ be the street network graph and $G=GraphReductionL1(\hat{G})$ the reduced graph obtained by applying the algorithm (Graph Reduction L1). Then the cost of the optimal solution calculated on graph *G* is less or equal to the cost of the optimal solution calculated on graph $\hat{G}$.

#### Proof

Given $\hat{S}$ a feasible solution of our problem on graph $\hat{G}$, let $\hat{R}$ be a sequence of nodes representing a generic route belonging to $\hat{S}$ associate with a generic vehicle $v \in V$. Removing from $\hat{R}$ all the nodes with null demand, we will get a new sequence of nodes in *R*. Considering that, by construction, between each pair of successive nodes in *R* there is a path in $\hat{G}, R$ is a route also in *G*. Given two consecutive nodes $(n_i, n_j)$ in *R*, in $\hat{R}$ there is the path composed of nodes $(n_i, \ldots , n_j)$. Since in *G* the cost of each arc is the minimum cost path between the same pair of nodes in $\hat{G}$, the cost $c_ {i j v}$ of the arc $(n_i,n_j)$ traveled with vehicle $v \in V$ is less than or equal to the cost of the route from node $n_i$ to node $n_j$ in $\hat{G}$, using vehicle $v \in V$; this means that the cost of route *R* will always be less than or equal to the cost of route $\hat{R}$.

Moreover, the length $l_ {i j v}$ of the arc $(n_i,n_j)$ traveled with vehicle $v \in V$ is less than or equal to the cost of the route from node $n_i$ to node $n_j$ in $\hat{G}$; this means that the length of route *R* will always be less than or equal to the length of route $\hat{R}$, respecting the maximum duration constraint. Considering also that between routes $\hat{R}$ and *R* the sum of the customer demand does not change, then the maximum capacity of vehicle $v \in V$ is never exceeded. With this simple transformation we have shown that it is possible to transform each solution $\hat{S}$ in $\hat{G}$ into a solution *S* in *G* with objective function value less than or equal to that of $\hat{S}$. To prove that it is possible to create a better solution in *G* than the optimal solution in $\hat{G}$, we will use the example in Fig. 3c. $\square$

## Mixed integer linear programming model

In order to model the distribution problem in urban areas defined in the previous session, we define the following decisional variables.

$$\begin{aligned} x_{ijv}&= {\left\{ \begin{array}{ll} 1 &{} \text {if vehicle }v \text {travels from }i \text { to }j \\ 0 &{} \text {otherwise;} \end{array}\right. } \\ y_{ijv}&\in [0,k_v],\text { representing the amount}\\&\text {of goods carried by vehicle }v \text {from }i \text {to} j. \end{aligned}$$

Let us now give the proposed MILP model of the problem that is derived from Cerulli et al. (2018). Note that the given MILP model refers to a decision planning problem of a single time slot within a working day.

3 $$\begin{aligned} MIN \ z=\sum _{v \in V} \sum _{(i,j) \in A}c_{ijv}x_{ijv} + \sum _{v \in V} \sum _{(d,j) \in A}s_{v}x_{djv} \end{aligned}$$

s.t.

4 $$\begin{aligned}&\sum _{v \in V}\sum _{i \in N}x_{ijv}=1 \, \forall \ j \in U \end{aligned}$$

5 $$\begin{aligned}&\sum _{i \in N}x_{ikv}-\sum _{j \in N}x_{kjv}=0 \, \forall \ k \in U, \ \forall \ v \in V \end{aligned}$$

6 $$\begin{aligned}&\sum _{i \in N}y_{ikv}-\sum _{j \in N}y_{kjv} =q_k \sum _{h \in N} x_{hkv} \, \forall \ k \in U, \ \forall \ v \in V \end{aligned}$$

7 $$\begin{aligned}&y_{ijv}\le (k_v -q_i)x_{ijv} \forall \ (i,j) \in A, \, \forall \ v \in V \end{aligned}$$

8 $$\begin{aligned}&y_{ijv} \ge q_jx_{ijv} \forall \ (i,j) \in A, \, \forall \ v \in V \end{aligned}$$

9 $$\begin{aligned}&\sum _{(i,j) \in A}l_{ijv}x_{ijv}+\sum _{(i,j) \in A: j \in U}L_cx_{ijv} \le L_v \, \forall \ v \in V \end{aligned}$$

10 $$\begin{aligned}&y_{ijv} \ge 0 \forall \ (i,j) \in A, \, \forall \ v \in V \end{aligned}$$

11 $$\begin{aligned}&x_{ijv} \in \{0,1\} \forall \ (i,j) \in A, \, \forall \ v \in V \end{aligned}$$

In the above model, the objective function (3) minimizes the total distribution cost to serve all customers, given by traveling, loading, and environmental cost components, as defined in Sect. 2.1, plus the setup cost associated with the activation of each vehicle. Constraints (4) impose that a customer is served exactly once by a vehicle, while constraints (5) impose that if a vehicle delivers goods to a customer, it must depart from it.

Constraints (6) are the so-called flow-commodity constraints, specifying the difference between the quantity of goods a vehicle carries before and after visiting a node. Constraints (7) guarantee that the capacity of the selected vehicle is never exceeded. Constraints (8) impose that the value $y_{ijv}$ must be at least equal to demand $q_j$ of node *i* if a vehicle *v* crosses the arc $(i,j) \in A$. Constraints (9) ensure that the maximum length of the route traveled by each vehicle *v* is no longer than a given value $L_v$. Finally, (10) and (11) define the decision variables.

To reduce the symmetry of the above model (3)–(11), for each class of vehicles *t* we added the following family of constraints (12) to the model. Suppose that in a given class there are *h* vehicles $\{v_1, v_2, \ldots , v_h\}$. For this class of vehicles we added $(h-1)$ anti-symmetry constraints. The aim is to avoid the generation of identical solutions which differ in the allocation of routes to different vehicles of the same class. In particular, to create these constraints, we associate a unique integer number *id*(*d*, *j*) to each outgoing arc from the depot.

12 $$\begin{aligned} \sum _{(d,j)\in A}id(d,j)x_{djv_i} {\ge } \sum _{(d,j)\in A}id(d,j)x_{djv_{(i+1)}}&\forall \ i \in [1,h] \end{aligned}$$

Constraint (12) allows us to create a route/vehicle assignment order. The ordering ensures that in the case of *h* equivalent vehicles, the route vehicle assignment will always be unique. This is true because thanks to our reduction algorithm (see Sect. 2.2), the arc leaving the depot is associated with the first customer served on the route, making the integer *id*(*d*, *j*) unique for each route.

## Heuristic approach

In our study, we present two different approaches to solve our GVRP problem: a math-heuristic approach and a memetic approach.

### Math-heuristic approach

The math-heuristic methods proposed in the literature to solve a different variant of the VRP could be classified in the following three classes (see the survey by Archetti and Speranza 2014).

- Decomposition approaches: the problem is split into smaller and simpler subproblems, and a specific solution method is applied to each subproblem.
- Improvement heuristics: use a mathematical programming model to improve a solution found by a different heuristic approach.
- Column generation-based approaches: Branch-and-price algorithms. Such algorithms use a set partitioning formulation by associating a binary variable to each possible route (column). Due to the exponential number of variables, the solution of the linear relaxation of the formulation is performed through column generation.

Our approach can be considered belonging to class 2 with a substantial modification: we designed the heuristic in order to give more importance to the optimization of the objective function at the expense of its feasibility. The restoration of the feasibility is entrusted to the mathematical model. In detail, our math-heuristic (MAT) approach uses the mathematical model to solve a “reduced” version of the instance to be solved.

For each instance to be solved, we perform the iterated local search (ILS) technique several times, using a multi-start approach, and analyze all the different solutions obtained. As a result of this process, we try to identify how often each arc is present or not in the generated solutions. In this way, we reduce the size of the instance by eliminating arcs that have a low probability of being part of the optimal solution and leaving (promising) arcs that have a good probability of being part of the optimal solution. To implement this reduction, for each arc, we count the number of times it is present in the final ILS solution: those arcs that exceed a predetermined threshold are left in the graph, while those below this threshold are eliminated. Obviously, this reduction process is carried out by preserving the connection of the graph. In the implementation of the proposed technique, It is chosen a dynamic threshold for each node, such that at least five incoming and five outgoing arcs are present for each node of the graph after the reduction.

### Memetic approach

An extensive review and analysis of the main meta-heuristics applied to the various VRP problems were presented in the paper by Vidal et al. (2013, 2019). The meta-heuristics used to solve VRP and which have given the best results are Tabu search, simulated annealing, deterministic annealing (Li et al. 2012), genetic algorithms (Minocha et al. 2011), ant colony (Donati et al. 2008), and variable neighborhood search implemented by Hemmelmayr et al. (2009).

The memetic approach we designed is obtained by embedding our simple local search (LS) in a genetic framework. In particular, we apply the LS to each chromosome that our GA produces. We use this memetic approach instead of a classic GA to guarantee the feasibility of the solutions generated. In fact, constraint (9) in the model (3)–(12), which limits the maximum length of each route, makes it very hard to get feasible solutions. For this reason, the crossover and mutation operators we designed are highly destructive and, in general, do not guarantee the feasibility of the solutions produced. Then, we combine the power of the GA that tries to improve the solutions without caring too much about the feasibility with the accuracy of the LS that looks to restore the feasibility.

**Fig. 4:**
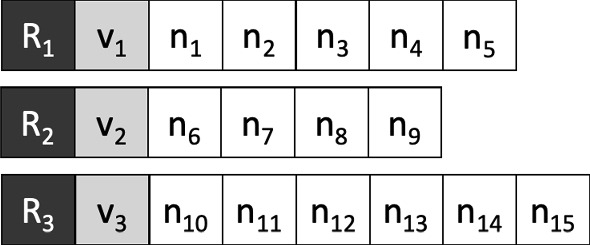
Representation of a generic solution

Each solution *S* is composed of a set of routes *R*. A vehicle and a sequence of nodes representing the path are associated with each route. The first element of each route is always the depot *d*. An example of a solution to our problem is reported in Fig. 4.

In the remainder of this paper, we denote $\mid S\mid$ the number of routes contained in a solution *S*, and we use $\mid R\mid$ to indicate the number of customers in the route *R*.

### Constructive algorithm

The purpose of this algorithm is to create an initial solution (not necessarily feasible) to be used as input for the successive approaches. Until all customers are not served, this algorithm: (i) creates an empty route; (ii) associates a vehicle with the route; (iii) adds other customers to the route until the vehicle has residual capacity. The route always starts from the depot. Then, iteratively, the customers are added, choosing among the not served customers one, close enough to the last customer reached. Note that this scheme does not take into account constraints (9) of the MILP model, while constraints (7) are considered in the construction phase of the solution. A detailed pseudo-code of the algorithm is shown in Algorithm 3.
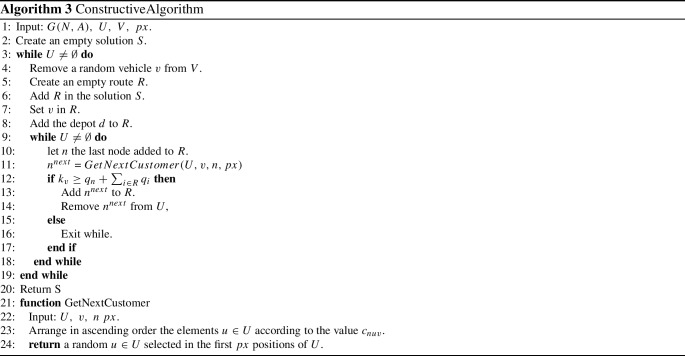


This algorithm creates a random solution *S*. As it is possible to notice for the schema reported in Algorithm 3, this solution depends on the parameter *px*. More precisely, with $px = 1$, we have a solution in which, for every route, the Nearest Neighbor policy is used. Increasing the value of *px*, we can get very different solutions from each other. Since this procedure takes into account only the capacity of the vehicle and not the maximum travel time of a route, it is possible to find unfeasible solutions to the problem. It would be easy to remedy the problem, but since the solutions produced are only useful as initial solutions for the algorithms proposed in the next sections, a degree of unfeasibility has proved to be experimentally useful to guarantee a wider exploration of the feasible region.

### Fitness function

In order to compare two solutions and determine the best, it is usually sufficient to use the objective function associated with the problem. Unfortunately, when algorithms have to compare solutions that are not always feasible, the use of the objective function alone is not enough. In fact, the objective function value may not be calculable in the case of an unfeasible solution, or this value may not be representative of the goodness of the solution.

In this paper, we use the objective function as a fitness function in the case of a feasible solution. In the case of an unfeasible solution, we add a penalty to the fitness function useful to represent the degree of the infeasibility of the solution.

**Fig. 5:**
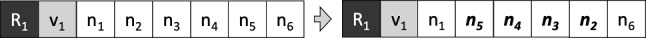
Example of neighborhood 2-OPT applied on the couple on arcs $(n_1,n_2),(n_5,n_6)$

**Fig. 6:**
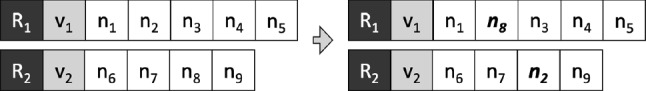
Example of neighborhood Swap, the customer $n_2 \in R_1$ is exchanged with the customer $n_8 \in R_2$

**Fig. 7:**
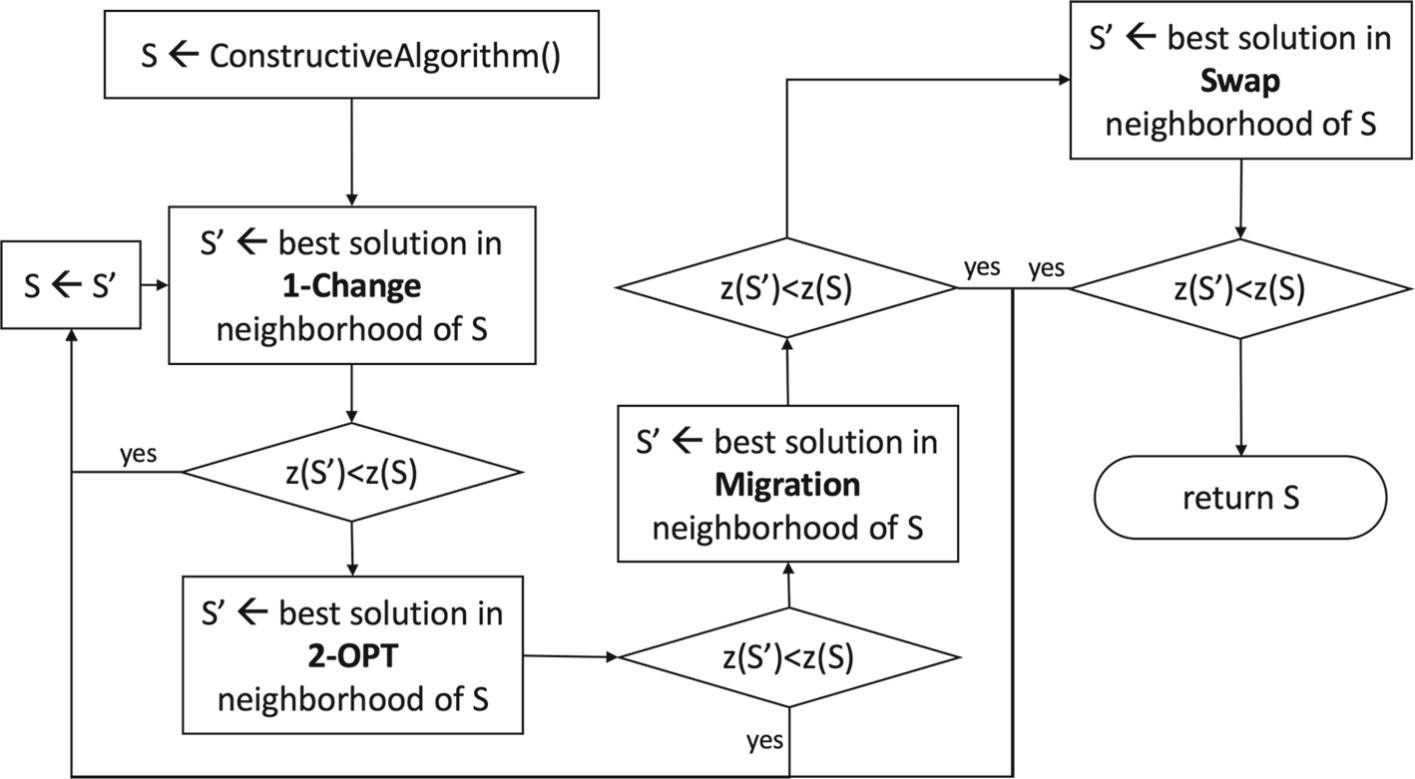
Local search (LS) algorithm flowchart

Given the solution *S*, $S_z$ represents the objective function value of the solution. In the case of an unfeasible solution, $S_6$ represents the total violation in terms of a vehicle’s maximum capacity (violation of constraint 6), and $S_8$ represents the total violation in terms of routes length (violation of constraint 8). The fitness function *ff* computed for solution *S* is equal to: $ff(S)=S_z+M+M_6S_6+M_8S_8$, where *M*, $M_6$ and $M_8$ are three penalty multipliers. For all three *M* penalty parameters in the experimental tests, we used the value 10,000.00.

### Local search

A local search algorithm was developed to make the solution proposed by the constructive approach feasible and improve its quality. The local search is a technique that makes use of the neighborhood of a solution. Starting from an initial solution, this is iteratively improved by exploring its neighborhood until the technique reaches a local minimum. In order to describe the implemented algorithm, it is necessary to introduce the neighborhood used. In our implementation, we used four different neighborhoods combined in sequence. Before describing the algorithm, we introduce the used neighborhood.

**Neighborhood 1: One-Change.** For each route $R \in S$, for each customer $n \in R$, *n* is removed from *R* and reinserted in *R* in every possible position. This neighborhood will therefore contain a number of solutions equal to $\mid S\mid *\mid R\mid *(\mid R\mid -1)$.

**Neighborhood 2: 2-OPT.** For each route $$ R \in S $$, for each couple of arcs $$ (n_i,n_j),(n_k,n_h) \in R $$, the arcs $$ (n_i,n_j)$$ and $$(n_k,n_h)$$ are removed from *R*, the path from $$n_j$$ to $$n_k$$ in *R* is reversed and the arcs $$ (n_i,n_k),(n_j,n_h)$$ are added to *R* to close the route. This neighborhood will therefore contain a number of solutions equal to $$\mid S\mid *\mid R\mid *(\mid R\mid -1)$$. Figure 5 shows an example of this operator.

**Neighborhood 3: Swap.** For each pair of routes $R_1, R_2 \in S$, for each pair of customers $n_1 \in R_1, n_2 \in R_2$, in the route $R_1$ the customer $n_1$ is replaced by $n_2$ and in the route $R_2$ the customer $n_2$ is replaced by $n_1$. This neighborhood will therefore contain a number of solutions equal to $\mid S\mid *(\mid S\mid -1)\mid R_1 \mid *\mid R_2\mid$. Figure 6 shows an example of this operator.

**Neighborhood 4: Migration.** For each pair of routes $R_1, R_2 \in S$, for each customer $n_1 \in R_1$, $n_1$ is removed from $R_1$ and added to $R_2$ in every possible position. This neighborhood will therefore contain a number of solutions equal to $\mid S \mid *(\mid S \mid -1) \mid R_1 \mid *\mid R_2\mid$.

The local search algorithm is summarized in the flowchart shown in Fig. 7.

### Genetic algorithm

Genetic algorithms are powerful metaheuristic algorithms, initially introduced by Holland (1975) and largely used to solve complex combinatorial optimization problems. Holland was inspired by Darwin’s theory of evolution, based on the idea of natural selection. Genetic algorithms somehow reproduce the mechanisms which characterize the evolution of life forms. Basically, they simulate the evolution of a population of individuals, each of which represents a feasible solution to the problem. The algorithm, starting with an initial population, performs a certain number of iterations, producing at each step a new generation of individuals created on the basis of the previous one. In this section, we describe a genetic algorithm for our problem. First of all, we need to specify what is the form of a solution, or rather how we encode the chromosome which represents a solution. Then, we can define the fitness function and the stopping criteria and describe how the selection, crossover, and mutation operations are performed.

**The chromosome**

The purpose of the chromosome is to represent a generic solution to the problem. The genetic algorithm uses the same representation of the solution introduced in Sect. 4 and shown in Fig. 4.

**The fitness function**

The fitness function is used to associate a numerical value to each chromosome. This value is essential to be able to compare the chromosomes. Usually, this function is a direct consequence of the objective function of the problem, but it can manage the unfeasibility of some solutions through an appropriate penalty. The genetic algorithm described in this work uses the fitness function presented in Sect. 4.4.

**The crossover**

**Fig. 8:**
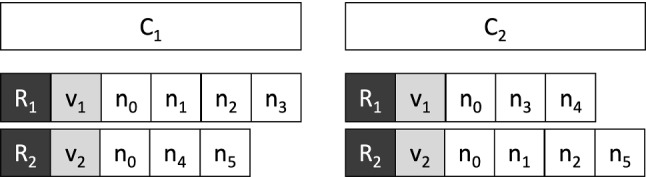
An example of input chromosomes for the crossover

**Fig. 9:**
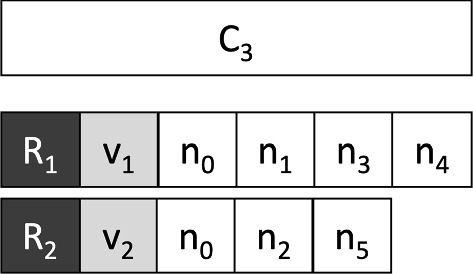
Crossover example associated with Table 2

In a genetic algorithm, the crossover is usually used to create individuals of generation $(i + 1)$ starting from some individuals of generation *i*. Our implementation of the crossover requires 2 chromosomes called parents and produces as output a new chromosome called son. Our crossover procedure is very simple; it starts from the parent chromosomes $C_1, C_2$ and creates the child chromosome $C_3$ by iterating over all the nodes of the graph excluding the depot. For each node $n_i$ the vehicle that will serve it in $C_3$ is chosen randomly between the vehicle that serves $n_i$ in $C_1$ or $C_2$. Figure 8 shows an example of input chromosomes for the crossover. Table 2 shows for each node, the vehicle that serves it in $C_1$ and $C_2$. Figure 9 shows the possible crossover resulting by choosing randomly from Table 2 a vehicle for each node.

**Table 2:**
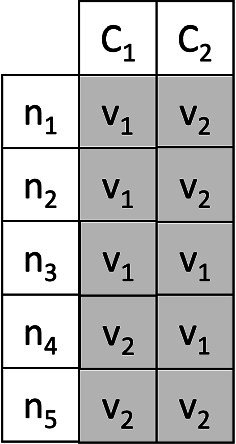
Random choice of the assignment of vehicles to each node

**The mutation**

The mutation procedure used is very invasive but, at the same time, elementary to describe. For $\sqrt{\mid N\mid }+1$ nodes, a new vehicle is randomly set among all those available. All routes associated with nodes for which the vehicle has been modified are shuffled. The reasons for using such a strong mutation will be explained later.

**Fig. 10:**
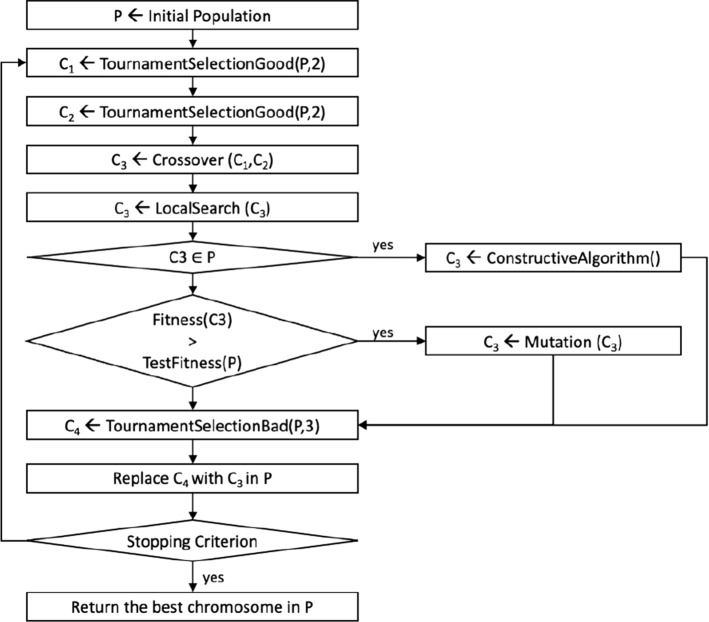
Synthetic scheme of the genetic algorithm

**The initial population**

The initial population is created by using the constructive algorithm (Algorithm 2) introduced in Sect. 4.3, setting the parameter $px = 5$. The population is composed of 100 chromosomes. The size of the population, as well as all the other parameters of the genetic algorithm, has been chosen following an intense manual tuning phase.

**The Genetic Algorithm scheme**

The GA starts from an initial population *P* created as described above. Then, two chromosomes $C_1$, $C_2$ are chosen using a tournament selection. The tournament selection “TournamentSelectionGood” requires as input the population *P* and an integer denoted *el*. The procedure randomly selects a number of chromosomes from *P* equal to *el*, and returns the one with the best fitness function. The next step involves creating the $C_3$ chromosome by applying the crossover operator. The chromosome $C_3$ is improved by applying the previously described local search algorithm. If $C_3$ is already present in the population, this chromosome is reconstructed using the same technique already used for the creation of the initial population. If $C_3$ is not present in the population, its fitness is compared to the median fitness of the population. If the fitness of $C_3$ is greater than the median fitness of the population, the mutation operator is applied to chromosome $C_3$. Otherwise, we proceed by selecting a chromosome to be removed from *P* to empty a slot for $C_3$. $C_4$ is the chromosome to be removed from the population. It is selected through the “TournamentSelectionBad” procedure which behaves extensively like the previous tournament selection except for the fact that this procedure chooses the chromosome with worst fitness value. The procedure restarts again until the stopping criterion is reached; otherwise, the chromosome in the current population whose fitness value is the best (the minimum) is returned. This genetic algorithm scheme has the characteristic of not making the population evolve for generations but of allowing a constant evolution of the current population. To forcefully introduce the concept of generation into the population, we can say that the population of generation *i* differs from that of generation $i + 1$ by exactly two chromosomes. The operating scheme of *GA* is summarized in Fig. 10. The extreme randomness of the operators used within this genetic algorithm is due to the presence of a very strong local search which could alternatively cause a rapid convergence of the population towards a local minimum.

**Table 3:** Computational results on medium-size instances

$\mid V\mid$	*Cl*	$\mid U\mid$	Model		LS		GA		MAT	
			Value	Time	Gap (%)	Time	Gap (%)	Time	Gap (%)	Time
2	2	10	42.10	0.4	0.0	0.0	0.0	0.7	0.0	0.2
4	2		49.58	3.6	0.0	0.0	0.0	0.7	0.0	0.3
3	3		41.78	0.6	0.8	0.0	0.4	0.6	0.0	0.2
2	2	15	51.85	21.8	0.0	0.0	0.0	3.3	0.1	0.5
4	2		49.72	69.9	0.1	0.0	0.0	2.8	0.2	1.3
3	3		50.03	36.7	0.0	0.0	0.0	2.8	0.2	0.7
2	2	20	54.41	1549.9	0.5	0.0	0.0	4.5	0.2	2.7
4	2		53.66	2017.0	0.9	0.0	0.0	4.8	0.1	8.3
3	3		52.78	349.8	0.3	0.0	0.0	4.9	0.3	4.6
4	2	25	75.58	3600.2	− 0.8	0.1	-1.0	9.0	-0.8	114.4
6	2		76.12	3600.9	− 3.4	0.0	-5.1	4.6	-4.5	166.1
6	3		73.14	3600.3	− 0.8	0.0	-1.3	4.8	-0.7	108.5
4	2	30	86.04	3600.8	− 9.8	0.1	-10.5	12.2	-10.0	279.9
6	2		79.00	3602.0	− 4.1	0.1	-6.4	9.5	-6.3	230.7
6	3		75.82	3601.5	0.5	0.1	-2.9	8.7	-2.6	212.4
4	2	35	104.52	3602.3	− **0.5**	0.2	-3.5	16.4	-0.8	600.7
6	2		110.35	3601.7	− 9.6	0.0	-10.9	10.7	-8.8	600.4
6	3		107.51	3602.3	− 7.1	0.0	-9.0	11.2	-4.6	600.5
4	2	40	**107.22**	3604.3	**No**	0.1	-4.6	25.6	-**2**.**8**	600.2
6	2		153.40	3601.3	− 35.9	0.1	-37.0	14.0	-30.4	601.0
6	3		150.98	3601.4	− 34.5	0.1	-37.3	18.6	-41.2	437.7
		**Avg**		2250.9	− 5.2	0.0	-6.1	8.1	-5.4	217.7

$\mid V \mid$ indicates the number of available vehicles, the number of vehicle classes is indicated by *Cl*, while $\mid U \mid$ indicates the number of customers. For the MILP model, both the value of the solution and the running time are shown. For the local search (LS), the genetic algorithm (GA) and the metaheuristic (MAT) in addition to the running time, the percentage of increase of the solution value with respect to the MILP model is reported. Times are shown in seconds. For the MILP and for MAT, the maximum running time is limited to 3600 and 600 s, respectively. The experiments for which a solution has not always been produced are highlighted in bold

## Computational experiments

In this section, we will report the experimental results of the tests performed. For these experiments, we used input instances of three types: (i) instances from paper (Cerulli et al. 2018), (ii) instances randomly generated, (iii) instances related to the city of Genoa. All the code has been implemented in Java, using CPLEX version 12.8 as a solver (with the default setting on all parameters and without thread limit). All tests were performed on a laptop with the following hardware features: MacBook Pro, processor Intel i9 2.9GHz, 32GB RAM. For all the experiments, we set the following operating scenario: 2 h time window, vehicle speed 40*Km*/*h*, 7 min service time. This scenario implied that $L_v=80Km$, $L_c=5Km$. The local search algorithm was iterated 100 times (the same value as the GA population), and the best objective function value and the sum of the running times of the 100 iterations is shown in the tables reported in the next subsections.

The first set of instances used for our experiments is described in the paper of Cerulli et al. (2018) and is representative of a real problem of distribution of goods within the city of Genoa. Thanks to our mathematical model, which makes use of the reduction phase of the input graph, we were able to solve optimally all the input instances in a computation time less than 1 s, compared to the minutes or hours needed for the model proposed in the original work. For this reason, we realized that in order to test the developed approach, larger instances had to be used. In the next sections, we will introduce these instances describing their characteristics.

### Random instances

This new set of instances was generated randomly; the motivation is that we wanted to carry out test cases useful to validate different characteristics of our algorithms. The approach used to create the instances can be schematized as follows: (i) place all customers within a $1000\times 1000$ size grid randomly; (ii) the distance between customers is set equal to the respective Euclidean distance; (iii) the cost of each arc is set equal to its length multiplied by a value in the range [0.216: 0.432] randomly chosen for each type of vehicle (see Fig. 1); (iv) the demand of each customer is set to a random value in the range [1, 10] ; (v) for each type of vehicle, the capacity is set randomly so that the sum of all the capacities of all the vehicles is double compared to the total customer demand. This leads to the generation of instances for which at least half of the vehicles must be selected. Two sets of instances were generated using this algorithm, referring, respectively, to medium and large case instances; the corresponding computational results are reported in Tables 3 and 4, respectively. For each scenario, five different instances have been created; the values reported are the average of the generated instances.

With reference to the medium size instance (see Table 3), the proposed model (3)–(12) is able to obtain the optimal solution in less than 1 h of CPU time in scenarios with up to 4 vehicles and a maximum of 20 customers. The LS algorithm is very fast and produces good solutions but, unfortunately, sometimes fails to identify a feasible solution (see the cases highlighted in bold in Table 3). Although the GA algorithm is computationally more expensive, it always finds a feasible solution, which is almost always the optimal solution; when the model fails to find the optimal solution, GA improves in a few seconds the results obtained by the model in 1 h. The math-heuristic in a computational time lower than the mathematical model produces good quality results. In two scenarios, MAT produces better solutions than GA.

In the case of large instances (see Table 4), the genetic algorithm produces very good quality solutions in a maximum CPU time of about 2 min. LS is very fast but, in cases where it is very difficult to find a feasible solution, often it remains trapped in an unfeasible local minimum without being able to provide a solution (see the three cases having the value “No” in Table 4). Since we noticed that the proposed LS algorithm is very fast and produces good solutions in only 100 iterations, as a further test, we wanted to verify what would happen by increasing this number to a higher running time than GA. The answer is that at 100,000 iterations, the LS becomes slower than GA and produces many unfeasible solutions (12), never reaching the quality of GA’s solution. These results are summarized in Table 5.

**Table 4:** Computational results on large size instances

$\mid V\mid$	*Cl*	$\mid U\mid$	LS		GA	
			Value	Time	Value	Time
6	2	50	133.4	0.1	130.5	29.8
8	2		131.6	0.1	128.6	24.2
9	3		133.7	0.0	126.4	34.2
6	2	60	**No**	0.1	150.7	49.0
8	2		151.9	0.1	143.2	40.5
9	3		149.9	0.1	142.4	55.4
8	2	80	**No**	0.2	170.6	107.8
10	2		189.4	0.2	177.2	99.4
12	3		187.5	0.1	169.7	99.4
10	2	100	**No**	0.3	226.7	120.8
12	2		231.5	0.2	221.4	134.5
12	3		228.7	0.2	213.3	113.5
		**Avg**	170.8	0.1	166.7	75.7

Refer to Table 3 for the meaning of the columns

**Table 5:** Gap between GA and LS increasing the iteration of the local search

iterations	Gap (%)	Time	#Inf.
1	17	0	27
10	9	0	17
100	6	0	14
1000	3	1	13
10,000	2	13	13
100,000	1	132	12

Using the instances shown in Table 4

### Genoa instances

This set of instances is composed of graphs extracted from the road network of the city of Genoa. To create the graphs, we used the data of OpenStreetMap contributors (2017). We generated directed graphs, taking into account the direction of the roads. For each vertex, we have the corresponding (x, y) coordinate in a 2D plane. Each arc of the graph is associated with its length on the actual street network. All the graphs are strongly connected. The generation of the instances uses the algorithm described in Capobianco et al. (2017) and used in Cerrone et al. (2019). The computational results on this set of instances are shown in Table 6. The complete graph of the instance of the city of Genoa consists of 8660 vertices and 16158 arcs. Note that this graph has been obtained by applying the graph reduction algorithm described in Sect. 2.2. From Table 6, readers can note that for scenarios up to about 20 customers, the proposed MILP model is able to find the optimal solution in an acceptable CPU time. The local search algorithm cannot get feasible solutions for the largest instances (6 vehicles, 63 and 66 customers), while the results reported in Table 6 confirm the good behavior of the GA algorithm. It is worth remembering that in all instances, in the graph representing the city of Genoa, the real distances of the arcs have been weighed not only with external costs due to environmental factors (see Sect. 2.1) but also with a penalty due to the transit of commercial vehicles near schools or other places of interest (see formula 2). For this reason, we wondered if the lower bound computed by using Proposition 3 (see Sect. 2.3) would produce a solution having the optimal value of the objective function lower than that found by the model (3)–(12) in the first three instances, for which we computed the optimal solution. Fortunately, the lower bound given by applying Proposition 3 gave the same solution for the three instances as the MILP model. This means that in these cases, the reduction algorithm did not remove the optimal solution by reducing the size of the graph.

**Fig. 11:**
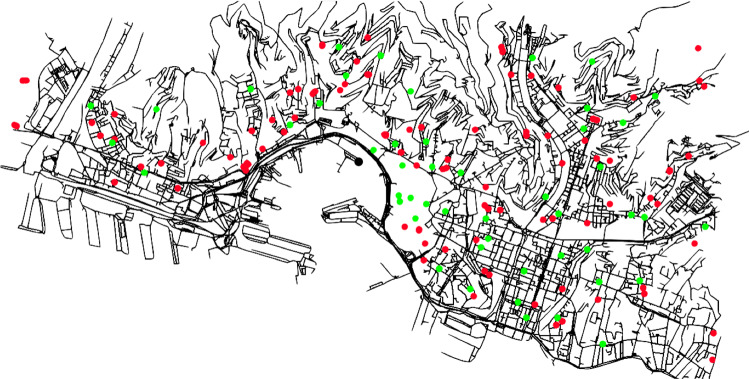
Graphical representation of the graph of Genoa

Figure 11 reports the graph of the city of Genoa. The green nodes represent the customers’ positions. The single black node represents the depot. The red points indicate the locations of the schools within the city. Before applying our algorithm to these instances, all the red nodes have been removed, and all the arcs within a distance less than 100*Mt* from these nodes have been marked as “not recommended” by setting the corresponding multiplier $\delta = 2$ instead of $\delta = 1$ for all other arcs.

**Table 6:** Computational results on the instances of Genoa

$\mid V \mid$	$\mid U \mid$	Model			LS		GA		MAT	
		LB	Value	Time	Value	Time	Value	Time	Value	Time
4	8	13.45	27.22	0.5	27.22	0.0	27.22	0.8	27.22	0.2
4	10	17.35	29.29	0.6	36.13	0.0	29.29	1.0	29.29	0.2
6	18	30.66	46.28	575.5	46.52	0.0	46.28	3.4	46.36	2.2
6	29	46.05	71.25	3601.8	71.84	0.0	63.70	7.0	64.23	73.6
6	31	57.28	78.42	3602.3	81.85	0.2	71.46	15.1	73.37	82.9
6	47	64.71	136.43	3600.7	106.35	0.1	104.94	24.5	106.78	600.8
6	63	68.88	No	3600.0	No	0.1	129.17	93.0	No	601.9
6	66	70.28	No	3600.0	No	0.1	131.01	55.9	No	600.5

$\mid V \mid$ indicates the number of available vehicles. For this experiment, we have two vehicle classes, LCV and HGV. $\mid U \mid$ indicates the number of customers. For the MILP model, for the local search (LS), for the genetic algorithm (GA), and the metaheuristic (MAT), both the value of the objective function and the running time are shown. Times are shown in seconds; for the MILP and for MAT, the maximum running time is limited to 3600 and 600 s, respectively. *LB* represents the value produced by the linear relaxation of the model

## Conclusion

In this paper, we focused on a variant of the Green Vehicle Routing Problem (*GVRP*) aimed at defining grocery distribution plans in urban areas, according to a vision of sustainable mobility, which is increasingly necessary today. In addition to the typical constraints of the problem, we included in the objective function both travel costs and externality costs due to environmental factors, which depend both on the type of vehicle used and the type of road crossed. Further, the arcs of the graph near schools, parks, or other places of interest have been associated with a penalty that increases their cost, thus encouraging the search for alternative routes. Finally, the delivery plans are defined considering only the time slots of a day in which in the city of reference is allowed the transit of commercial vehicles; in particular, from 2 to 4 h time slots are considered, including the service time. We presented a MILP model and two heuristic approaches, a math-heuristic and a memetic algorithm, both using a local search procedure. In particular, we imposed a constraint that limits the maximum length of a tour traveled by any vehicle to face two different issues on the urban freight distribution: the transit of commercial vehicles in the city and, in this period of the COVID-19 pandemic, the possibility of sanitizing the vehicle (and drivers) several times during the day. The reported computational experiments based on random instances specifically tailored for the problem show the efficiency of the proposed solution method. The validation of the proposed algorithms performed with the graph of the city of Genoa allows us to say that the proposed algorithms are a valuable aid in the definition of sustainable distribution plans in urban areas, also considering that the proposed model is easily adaptable to different urban realities and mobility rules.

In the future, we would like to investigate the proposed math-heuristic approach further. In particular, we want to make the mathematical model “tighter” and efficient and want to deepen the ways of reducing the size of the input graph based on the solutions proposed by the local search procedure.

## Data Availability

The datasets analyzed during the current work are available from the corresponding author on reasonable request.

## Declarations

### Code availability

Code developed for the model will be made available from the corresponding author on reasonable request.
